# Protection of outbred mice against a vaginal challenge by a *Chlamydia trachomatis* serovar E recombinant major outer membrane protein vaccine is dependent on phosphate substitution in the adjuvant

**DOI:** 10.1080/21645515.2020.1717183

**Published:** 2020-03-02

**Authors:** Sukumar Pal, Salvador Fernando Ausar, Delia F. Tifrea, Chunmei Cheng, Scott Gallichan, Violette Sanchez, Luis M. de la Maza, Lucian Visan

**Affiliations:** a Department of Pathology and Laboratory Medicine, University of California, Irvine, CA, USA; b Bioprocess Research and Development, Sanofi Pasteur, Toronto, Ontario, Canada; c Analytical Research and Development Department, Sanofi Pasteur, Toronto, Ontario, Canada; d Research & Non Clinical Safety Department, Sanofi Pasteur, Marcy l'Etoile, France

**Keywords:** Chlamydia trachomatis, subunit vaccine, neutralizing antibody, outbreed CD-1 mice, aluminum hydroxide adjuvant, recombinant major outer membrane protein, IFN-γ

## Abstract

*Chlamydia trachomatis* is the most common bacterial sexually-transmitted pathogen for which there is no vaccine. We previously demonstrated that the degree of phosphate substitution in an aluminum hydroxide adjuvant in a TLR-4-based *C. trachomatis* serovar E (Ser E) recombinant major outer membrane protein (rMOMP) formulation had an impact on the induced antibody titers and IFN-γ levels. Here, we have extended these observations using outbreed CD-1 mice immunized with *C. trachomatis* Ser E rMOMP formulations to evaluate the impact on bacterial challenge. The results confirmed that the rMOMP vaccine containing the adjuvant with the highest phosphate substitution induced the highest neutralizing antibody titers while the formulation with the lowest phosphate substitution induced the highest IFN-γ production. The most robust protection was observed in mice vaccinated with the formulation containing the adjuvant with the lowest phosphate substitution, as shown by the number of mice with positive vaginal cultures, number of positive cultures and number of *C. trachomatis* inclusion forming units recovered. This is the first report showing that vaccination of an outbred strain of mice with rMOMP induces protection against a vaginal challenge with *C. trachomatis.*

## Introduction

*Chlamydia trachomatis* is the most common sexually-transmitted bacterial infection worldwide.^1^,^2^ Genital infections affect mainly sexually-active teenagers.^3-5^ Newborns can become infected in the birth canal and contract ocular, respiratory and gastrointestinal infections.^6-8^ Trachoma, a chronic *C. trachomatis* ocular infection, affecting regions with poor sanitary conditions, is the most common cause of preventable blindness globally.^9-11^

The clinical sequelae, such as trachoma and pelvic inflammatory disease, are thought not to be directly caused by the pathogen itself, but by induced immune responses, which can clear the infection but can also cause collateral damage.^9,12,13^ *C. trachomatis* attracts innate and antigen-specific cells that release immune factors, such as chemokines and cytokines, which are likely to be responsible for the pathology. Results from studies in mice suggest that this pathogenesis is dependent on signaling through toll-like receptor 2 (TLR2), but not TLR4, although both TLR2- and TLR4-deficient mice can clear the infection as well as wild-type mice.^14^ Studies in humans and mice have shown that TLR2 is the primary pathogen-recognition receptor in the upper genital tract that drives the immune-pathogenic mechanisms associated with *C. trachomatis* infections.^14,15^

Many developed countries run *C. trachomatis* screening programs, although there are gaps in the evidence base for their efficacy.^16^ The European Center for Disease Prevention and Control (ECDC) recommends national strategies for *C. trachomatis* control, which include screening programs for at-risk individuals and an evaluation plan for the strategy. Antibiotic therapy is effective against *C. trachomatis* infections, but due to inappropriate treatment and the large number of asymptomatic patients, long-term sequelae including abdominal pain, infertility, ectopic pregnancy, lymphogranuloma venereum and blindness, can occur.^4,5,11,17^ The prevalence rate of genital infections has been reported to increase in countries that have established screening programs.^18,19^ This increase is thought to be due to reduced duration of infection, resulting from antibiotic therapy, which prevents the development of natural immunity.^18^ Therefore, vaccination could be the best approach to prevent *C. trachomatis* infections and reduce the burden of disease.^20-25^ The major outer membrane protein (MOMP), one of the most abundant, immunogenic chlamydial proteins, has been shown to elicit protective immunity in mice, similar to that induced by natural infection.^26^

Both innate and adaptive immune responses are elicited by natural *C. trachomatis* infections, with IFN-γ and IL-17 playing important roles as key effectors for both protection and pathological responses.^27^ At high concentrations, IFN-γ kills the bacteria but at low concentrations, it can enhance pathological responses.^12,13^ Hence, effective vaccines need to strike the correct balance between inducing a protective response and triggering a pathological reaction.

Phosphate substitution in aluminum-based adjuvants has been reported to modify the immunogenicity of various antigens in vaccine formulations. For example, in an anthrax vaccine containing recombinant protective antigen (rPA) and an aluminum oxyhydroxide AlOOH adjuvant, it was reported that high phosphate substitution was associated with a higher immune response to rPA.^28^ The authors suggested that this was due to a weaker interaction between rPA and the phosphorylated adjuvant that allowed a more complete water layer between the components and therefore resulted in a rapid release of rPA after injection.

Phosphate substitution in AIOOH-containing adjuvant can affect the adsorption and stability of vaccine components which can have an impact on the bioavailability of antigens, TLR agonists and thus the vaccine’s efficacy.^29-31^ This phosphate substitution can be achieved by pretreating the AIOOH-containing adjuvant with varying concentrations of phosphate ions in the form of potassium or sodium phosphate.^30,31^ The phosphate ions displace the surface hydroxyl thereby decreasing its point of zero charge. We recently reported that the cellular and humoral immune responses to a vaccine containing recombinant *C. trachomatis* serovar E MOMP, and a proprietary adjuvant (SPA08), composed of AIOOH and the TLR4 agonist, E6020, were dependent on the degree of phosphate substitution on the AlOOH component.^32^

The use of inbred strains of mice has greatly facilitated initial testing of experimental vaccine formulations by providing invaluable data on the assessment of the immune responses of the host which can help improve the protective efficacy of vaccine formulations. Inbred animals only provide information on a limited range of the possible immune responses observed in humans following vaccination. The high incidence and prevalence of chlamydial infections in all parts of the world affecting individuals with very different genetic backgrounds requires vaccine formulations to be tested in outbred individuals.

The initial studies assessing protection against trachoma were performed in various models, including humans and non-human primates.^11^ Most of the work done recently to assess the protective efficacy of vaccine formulations against genital infections utilized inbred mice, in particular C3H/HeN, BALB/c and C57BL/6 mice.^33^ Currently, one of the most extensive vaccine studies in an outbred species of animals is being performed in koalas (*Phascolarctos cinereius*).^34^ These animals that are naturally infected in the eyes and the genitourinary tract by *Chlamydia pecorum*, suffer long-term sequelae including blindness and infertility, like humans and therefore major efforts are underway to protect them with a vaccine using MOMP as the antigen.^35-38^

In the context of preclinical development of a human *C. trachomatis* vaccine, we focused on serovar E rMOMP as a model antigen since serovar E is the most prevalent serovar in human urogenital infections and also the one that induces the highest serological responses.^39-42^ Here, we report how phosphate substitution affected the ability of the rMOMP vaccine to protect outbreed CD-1 mice against a vaginal challenge with *C. trachomatis* serovar E. This was achieved using vaccine formulations with different degrees of phosphate substitution on the AlOOH-containing adjuvant, mentioned above, and another formulation containing aluminum phosphate, to represent a high degree of adjuvant phosphate substitution.

## Materials and methods

### C. trachomatis *stocks*

Stocks of *C. trachomatis* serovar E (strain Bour) were prepared in Hela-229 cell culture (American Type Culture Collection, Manassas, VA). Elementary bodies (EBs) were purified using Renografin and frozen at −70°C in SPG (0.02 sucrose, 0.2 M sodium phosphate, pH 7.2, and 5 mM glutamic acid).^43^ The titer of the EB stocks was determined using HeLa-229 cells.^43^

### Vaccine formulations

The recombinant *C. trachomatis* serovar E (strain Bour) MOMP (Ser E rMOMP) was expressed in *Escherichia coli* as inclusion bodies (IBs). Briefly, bacteria were grown in a 20-L fermenter with constant agitation at 37°C, pH 7 and 30% dissolved oxygen. The IBs were extracted and solubilized in 6M guanidine hydrochloride and then exchanged with 8M urea using tangential flow filtration (TFF). The urea solution was applied to an anionic exchange chromatography column (Q Ceramic HyperD® 20 Chromatography Sorbent, Pall Corporation) and Ser E rMOMP was eluted from the column with 50–90 mM sodium chloride. The eluted Ser E rMOMP was refolded by mixing with equal volumes of 10% v/v N-lauroyl sarcosine (NLS), 1 M 1-arginine and dithiothreitol (DTT) to a final concentration of 10 mM. This mixture then underwent buffer exchange with 10 mM Tris-HCl pH 8, 0.06% NLS diafiltration buffer using TFF and a membrane filter with a molecular weight cutoff of 10 kDa. The preparation underwent a second TFF with 50 mM Tris-HCl, pH 8.0 diafiltration buffer. The final purity of the Ser E rMOMP was greater than 90%, as evaluated by SDS-PAGE.

Aluminum hydroxide adjuvant (AlOOH) and aluminum phosphate adjuvant (AlPO_4_) were acquired from Brenntag (Frederikssund, Denmark). The SPA08 adjuvant was composed of the TLR4 agonist, E6020 (Eisai, USA), adsorbed onto AlOOH. Three SPA08 adjuvanted formulations containing 10 µg of Ser E rMOMP and 40 μg/mL E6020, 2.4 mg/mL elemental Al and no phosphate (SPA08-1), 88 mM (SPA08-3) or 176 mM (SPA08-4) phosphate were prepared as described previously.^32^ The phosphate to aluminum molar ratio (P:Al) of each adjuvant formulation is reported in Table 1. The SPA08-2 formulation, with 8.8 mM phosphate, was not assessed in this study as its immunological characteristics were previously shown to be similar to those of SPA08-1. An aluminum phosphate-based formulation (APF) with a high degree of phosphate substitution (APF/rMOMP) containing 10 µg of Ser E rMOMP was used for comparison.

**Table 1.** Physicochemical characteristics of Ser E rMOMP-SPA08 formulations

Adjuvant	P:Al molar ratio	Zeta potential (mV)	Size (d50) (µm)	% Adsorption	
				E6020	Ser E
SPA08-1	0	20.0 ± 1.1	4.6 ± 0.0	100.0 ± 0.0	100.0 ± 0.0
SPA08-3	1	−19.7 ± 3.4	4.0 ± 0.3	100.0 ± 0.0	68.9 ± 16.6
SPA08-4	2	−29.9 ± 6.4	4.8 ± 0.0	100.0 ± 0.0	44.2 ± 13.3
APF	NA	−33.0 ± 2.4	2.6 ± 0.1	100.0 ± 0.0	41.9 ± 0.2

### Physicochemical characterization of formulations

The zeta potential, particle size distribution and percentage of MOMP adsorbed for the three SerE MOMP-SPA08 formulations were determined as previously described.^32^

### Mice

Six-week-old outbred female CD-1 mice (Charles River Laboratories) were housed at the University of California, Irvine (UCI) in isolation cubicles at a constant temperature of 24°C with a 12 h light/12 h darkness cycle and fed chow and water *ad libitum*. The UCI Animal Care and Use Committee approved the animal protocols.

### Vaccination protocol

Vaccine formulations containing *C. trachomatis* Ser E MOMP and SPA08 or APF were extemporaneously prepared in 3 ml glass vials by mixing one volume of Ser E rMOMP stock solution with one volume of the SPA08-1, SPA08-3, SPA08-4 or APF adjuvants. The formulations were mixed manually by inverting the container at least five times to ensure thorough mixing before injecting the mice or conducting physicochemical characterization.

**Figure 1.**
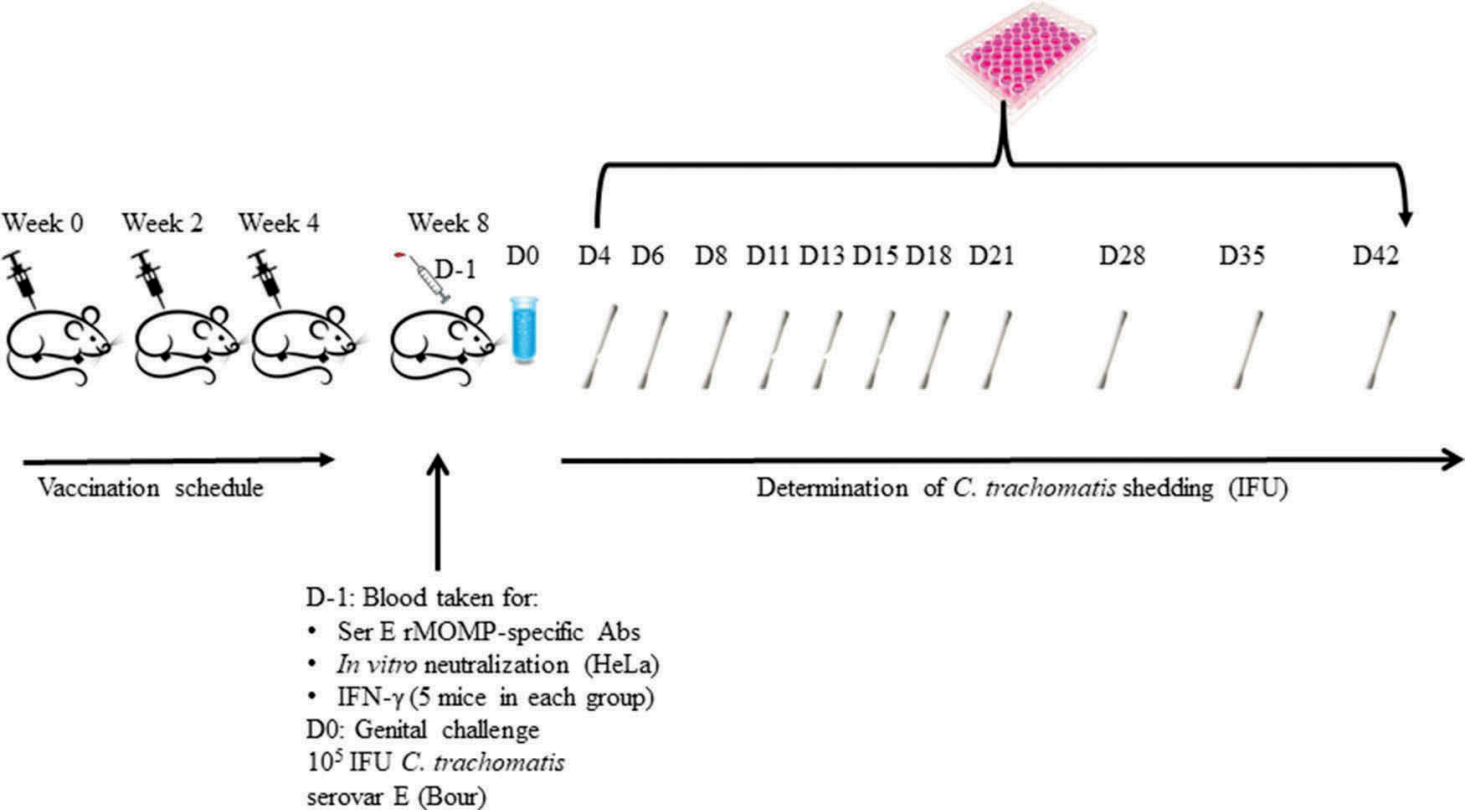
Schematic representation of study design

Groups of mice were vaccinated intramuscularly (IM) three times at 3-week intervals with 50 µL of the SPA08-1, SPA08-3, SPA08-4 or APF formulations (Figure 1). For each formulation, a group of mice was sham-vaccinated at the same time with the same volume of inoculum containing non-phosphate treated SPA08 or APF in phosphate buffered saline (PBS) with no Ser E rMOMP.

### *ELISA for* C. trachomatis *Ser E rMOMP-specific antibodies*

Blood was collected for IgG measurements and sero-neutralization assays by periorbital puncture the day before the vaginal challenge. *C. trachomatis* Ser E MOMP specific IgG, IgG1, IgG2a titers were measured by an enzyme-linked immunosorbent assay (ELISA) as described previously.^32,44^ Briefly, 96-well plates were coated with EBs in PBS at a concentration of 10 µg per ml (1 μg/well), and blocked with PBS-Tween-milk (0.05% Tween, 1% (w/v) powdered skim milk). The following steps were then carried out in a final volume of 100 µL, followed by three washes with PBS-Tween. Serial 2-fold dilutions of serum samples in PBS-Tween-milk starting from 1/1,000, or 1/10,000, were incubated at 37°C for 1 h. After washing the wells, horseradish peroxidase-conjugated goat anti-mouse IgG, IgG1 or IgG2a (BD Pharmingen, San Diego, CA) were added and the plates incubated for 1 h at 37°C. After washing, 2,2ʹ-azinobis (3-ethylbenthiazoline-6-sulfonic acid) (ABTS) was added to the wells and the plates incubated at 37°C for 1 h. The optical density at 405 nm in the wells was measured in an ELISA plate reader (Bio-Rad Laboratories, Richmond, CA).

### In vitro *sero-neutralization assay*

*In vitro* sero-neutralization assays, performed in 96-well plates as described previously, were repeated three times on different days.^32^ Briefly, two-fold serial dilutions of mouse sera in Ca^2+^- and Mg^2+^-free PBS, pH 7.2, supplemented with 5% guinea pig serum were incubated with *C. trachomatis* Ser E [1x10^4^ infectious forming units (IFU)] for 45 min at 37°C. The mixtures were added to HeLa-229 cell monolayers, centrifuged for 1 h and incubated at 37°C for 48 h. After washing, the monolayers were fixed with methanol and stained with an in-house monoclonal antibody E4 to MOMP.^44^ The titer of a sample was the dilution that yielded 50% neutralization relative to the negative control serum from sham-vaccinated mice.

### Cell-mediated immune responses: IFN-γ determination

To assess T-cell memory responses, cytokine production was measured in splenic T-cells from five mice per group euthanized the day before the challenge.^44,45^ In brief, T-cell-enriched splenocytes (10^5^ cells/well) were co-cultured with antigen presenting cells (APCs; 1.25 x 10^5^/well), prepared by irradiating syngeneic, unseparated splenocytes from the harvested spleens (3,000 rads; ^137^Cs), and then incubated for two hours with either UV-inactivated *C. trachomatis* Ser E EB at a 1:1 ratio or purified Ser E rMOMP. Concanavalin A was included as a positive control. The supernatants were harvested and stored at −80°C until used for the determination of the IFN-γ concentrations by ELISA (BD Pharmingen, San Diego, CA; Labsystem Multiscan; Helsinki, Finland).

### Vaginal challenge

Four weeks after the third vaccine dose the mice were challenged vaginally.^46-49^ The mice were injected with 2 mg of DepoProvera subcutaneously twice prior to the vaginal challenge (days −10 and −3) to synchronize their estrus cycle in diestrus. The mice were anesthetized and *C. trachomatis* Ser E (Bour) (10^5^ IFU) were inoculated into their vaginas. The mice were kept in a recumbent position for 10–15 min after the challenge.

Vaginal swabs were collected and cultured twice weekly for the first three weeks, and then once-a-week in weeks 4, 5, and 6 after the vaginal challenge (Figure 1).^47^ The swabs were vortexed in 200 μl of sterile SPG and two aliquots from each specimen (100 μl and 10 μl) were inoculated into HeLa-229 cell monolayers in 48-well plates by centrifugation at 1,000xg for 1 h at room temperature. Following incubation at 37°C for 48 h, the chlamydial inclusions were stained with an in-house monoclonal antibody (E4) to MOMP.^50^

The following criteria were used to determine protection: number of mice with positive vaginal cultures, number of positive vaginal cultures over total number of cultures collected, number of *C. trachomatis* IFU recovered and length of time of vaginal shedding.

### Hydrosalpinx formation

Seven weeks after the vaginal challenge mice were euthanized and their genital tract macroscopically investigated to determine the presence of hydrosalpinx which is an indicator of upper genital tract pathology.^51^

### Statistical analyses

Using a computer model, it was shown that even a vaccine with 50% efficacity would have a significant impact on the clearance of *Chlamydia* from a defined community.^20^ Thus, the sample size was based on a criterion of at least 50% efficacity. The number of mice included in the experiments was based on previously published recommendations.^52^

Student’s *t* test, Fisher’s exact test and Mann-Whitney *U* test were used for analyses performed using SigmaStat (version 3.5) software. Differences were considered statistically significant when the *p*-values was <0.05.

## Results

### Characterization of vaccine formulations

The SPA08 adjuvant was treated with phosphate ions to increase the phosphate substitution on the AlOOH component to investigate the impact of Ser E rMOMP adsorption on the immunogenicity and protection of the vaccine. The impact of type of aluminum salt and adsorption on immunogenicity and protection was also evaluated using an aluminum phosphate version of the adjuvant (APF), with identical amounts of Ser E rMOMP. The physicochemical characterization of the SPA08 formulations indicated that the zeta potential at neutral pH decreased as the P:Al molar ratio increased, but there was no effect on the particle size or E6020 adsorption (Table 1). The percentage of Ser E rMOMP adsorption to AlOOH in the formulation at neutral pH decreased as the phosphate substitution increased primarily due to high electronegativity of the SPA08 adjuvant at high P:Al ratios which decreased the electrostatic interaction between Ser E rMOMP and SPA08 at neutral pH (Table 1). In contrast, E6020 remained bound to AlOOH, irrespective of the amount of phosphate substitution.

The physicochemical characteristics of the formulation containing APF adjuvant were similar to those of SPA08-4, with a strongly negative zeta potential and comparable Ser E rMOMP percentage adsorption (Table 1). However, the particles in the APF formulation were significantly smaller than those in the SPA08-adjuvanted formulations.

### *Association of P:Al molar ratio with total* C. trachomatis *Ser E rMOMP-specific IgG levels and their neutralizing capacity*

#### Ser E rMOMP-specific antibody response

**Figure 2.**
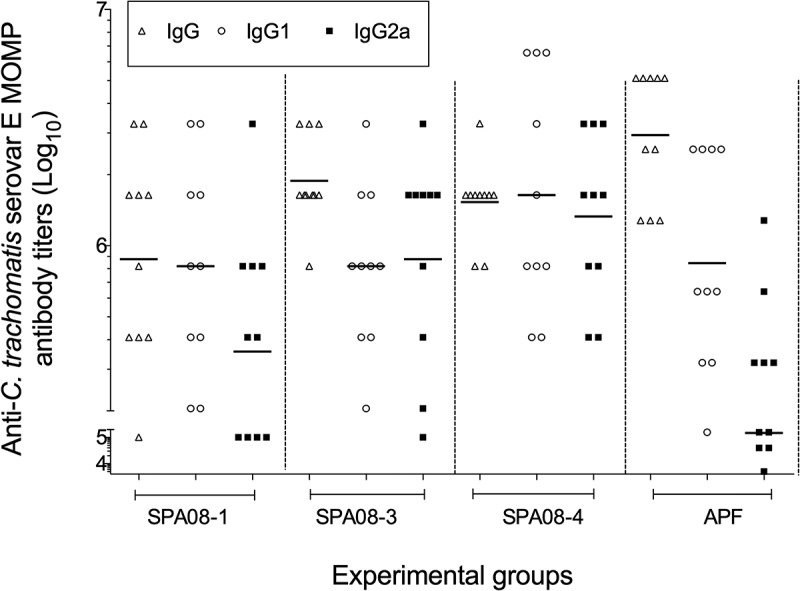
*C trachomatis* rMOMP serovar E-specific antibody titers determined by ELISA. Serum samples were drawn four weeks after the third vaccine dose from 10 mice. Each dot represents the results for one mouse and the horizontal lines represent the geometric mean titer (GMT) for the group of mice

High IgG titers in serum were observed in the four groups of mice immunized with *C. trachomatis* Ser E rMOMP independently of the adjuvant formulation used (Figure 2). Although mice immunized with SPA08-1 had lower IgG geometric mean titers (GMTs) (877,997; range 398,099–1,936,000) than those immunized with SPA08-3 (1,882,000; range 1,375,000–2,575,000), SPA08-4 (1,529,000; range 1,153,000–2,027,000) or APF (2,940,668; range 1,280,000–5,120,000) the differences were not statistically significant.

The IgG2a GMTs increased as the P/Al molar ratios increased (Figure 2). Similar IgG1 GMTs were elicited by SPA08-1 and SPA08-3, while the IgG1 GMTs elicited by SPA08-4 and APF were higher. The IgG1/IgG2a ratio for APF was 5.7 compared with a range from 0.93 to 2.3for the SPA08 formulations.

### In vitro *sero-neutralization assay*

**Figure 3.**
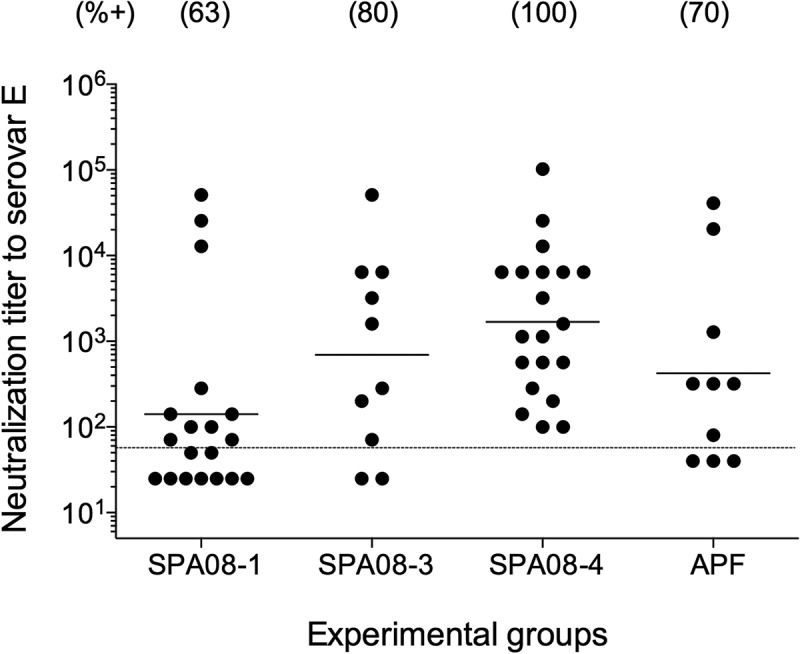
*In vitro* neutralization titers in sera drawn four weeks after the third vaccine dose. Each dot represents one mouse and the horizontal line indicates geometric mean titers (GMT)

Serum antibodies were evaluated for their ability to neutralize *C. trachomatis* Ser E infectivity *in vitro* (Figure 3). There was a trend for more mice to have neutralizing antibodies as the P:Al molar ratio increased: 12/19 (63%) in the SPA08-1 group; 8/10 (80%) in the SPA08-3 group and 20/20 (100%) in the SPA08-4 group. Positive neutralizing titers were observed in 7/10 (70%) of the mice in the APF group. The neutralizing GMTs were 141 (range: 25–51,200), 397 (range: 25–51,200), 1,685 (range: 100–102,400), and 422 (range: 40–40,960) for the SPA08-1, SPA08-3, SPA08-4, and APF groups, respectively.

### Association of P:AL molar ratio with ser E rMOMP-specific IFN-γ production

Ser E MOMP-specific cellular responses were tested by *ex vivo* stimulation of primed spleen T-cell with either Ser E EBs (*Ct*-E EB) or purified Ser E rMOMP. T-cell stimulation with purified Ser E rMOMP led to more IFN-γ production than stimulation with EBs for all three SPA08 groups (Table 2). The IFN-γ levels were higher for the SPA08-1 and SPA08-3 groups than those in the SPA08-4 group for both type of stimuli. The primed spleen cells from APF-immunized mice secreted the lowest quantities of IFN-γ when stimulated with either Ser E EB or rMOMP (Table 2).

**Table 2.** IFN-γ levels from stimulated spleen T-cells taken four weeks after the third vaccination from five mice in each group

Groups		IFN-γ levels (pg/ml, mean ± 1 SE) in response to:			
		Ct-E EB	Ct-E-rMOMP	Concanavalin A	Medium
SPA08-1	Vaccinated	7,538 ± 817^a^	15,563 ± 2,122^a^	36,351 ± 5,933	<15
	Control	<15	<15	19,311 ± 2,492	<15
SPA08-3	Vaccinated	2,748 ± 450^a^	11,206 ± 404^a^	45,599 ± 4,429	<15
	Control	<15	<15	43,226 ± 3,335	<15
SPA08-4	Vaccinated	1,814 ± 1,837^a^	5,416 ± 1,815^a^	15,848 ± 1,669	<15
	Control	<15	<15	26,045 ± 2,965	<15
APF	Vaccinated	1,679 ± 265^a^	1,166 ± 455^a^	37,593 ± 1,558	<15
	Control	<15	<15	48,682 ± 1,284	<15

^a^P<0.05 by the Student’s *t* test compared to corresponding PBS control group.

### Culture results following vaginal challenge

**Figure 4.**
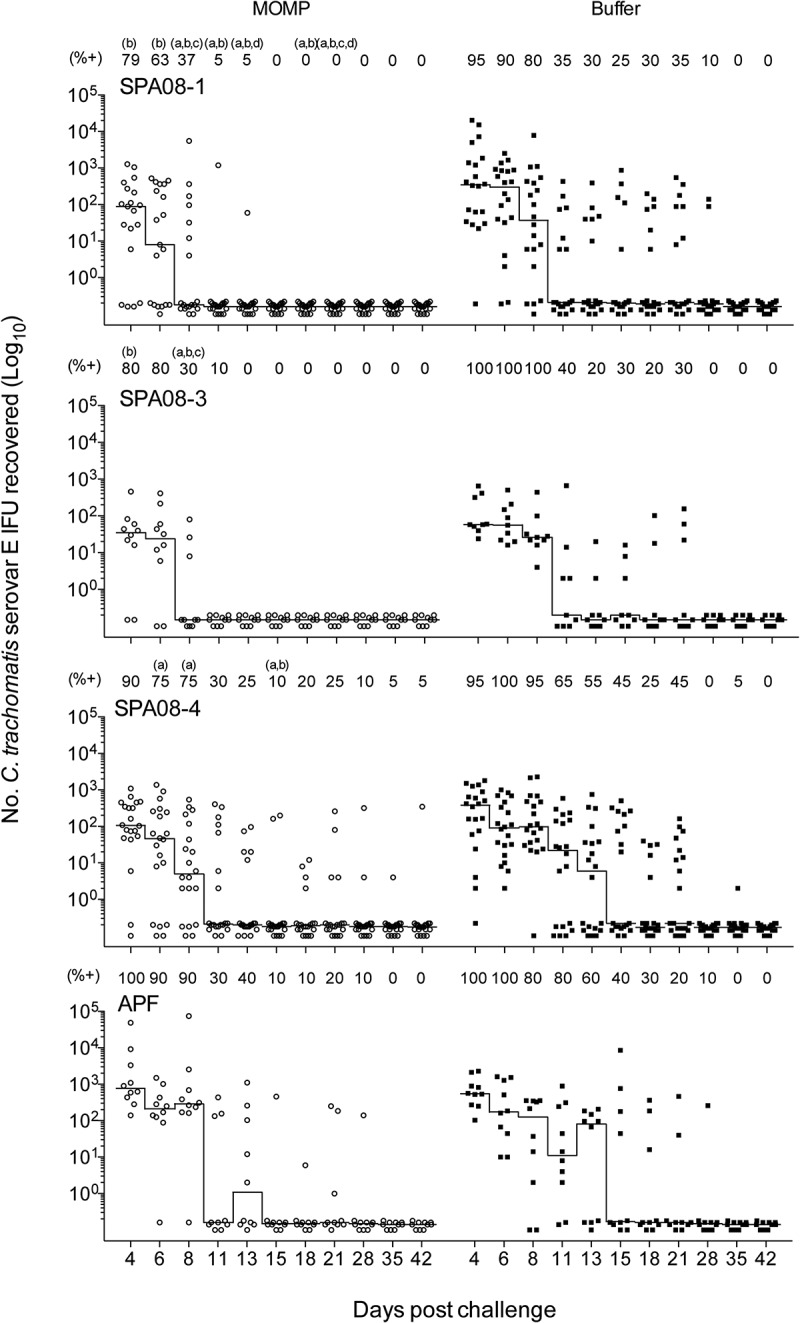
Vaginal culture results in samples taken at 4, 6, 8, 11, 13, 15, 18, 21, 28, 35 and 42 days after vaginal challenge with *C. trachomatis* Ser E

Vaginal swabs were collected from the CD-1 mice following the vaginal challenge twice weekly for the first three weeks and weekly thereafter. There were fewer mice with at least one positive vaginal culture in the rMOMP/SPA08-1 and rMOMP/SPA08-3 groups compared with their respective sham-vaccinated control groups, but not in the rMOMP/SPA08-4 group, however, the differences were not statistically significant (Figure 4; Table 3). The median number of days to negative culture was significantly shorter in the vaccinated groups compared with the sham-vaccinated control groups, except for the rMOMP/APF group. Infection was cleared faster in the rMOMP/SPA08-1 and rMOMP/SPA08-3 groups than in the rMOMP/SPA08-4 and rMOMP/APF groups. Moreover, there was no difference in the range of number of days to negative culture between rMOMP/APF and its sham-vaccinated control group. Significant differences in the median number of IFUs recovered/mouse were observed for the SPA08-1 and SPA08-4 groups and their respective sham-vaccinated control groups, but not for the SPA08-3 and APF and their sham-vaccinated control group. The mice in the APF immunized group were not protected compared with the mice in the sham-immunized group.

**Table 3.** Vaginal culture results from mice challenged vaginally with *C. trachomatis* Ser E

Vaccines	Mice with positive cultures/total (%)	Median no. days to negative culture (range)	No. of positive cultures/total (%)	IFU shed/mouse median (range)
rMOMP/SPA08-1	16/19 (84)^d^	8 (4–15)^a^	36/209 (17)^b^	413 (0–6,186)^a^
PBS/SPA08-1	19/20 (95)	11 (4–35)	86/220 (39)	1,504 (0–21,808)
rMOMP/SPA08-3	8/10 (80)^d^	8 (4–13)^a^	20/110 (18)^b^	118 (0–1,662)^c^
PBS/SPA08-3	10/10 (100)	13 (11–28)	41/99 (41)	180 (100–1,805)
rMOMP/SPA08-4	20/20 (100)^d^	12 (8–42)^a^	74/220 (34)^b^	235 (2–3,272)^a^
PBS/SPA08-4	20/20 (100)	18 (8–42)	106/220 (48)	1,052 (302–5,542)
rMOMP/APF	10/10 (100)^d^	15 (11–35)^c^	41/110 (37)^d^	1,971 (377–76,675)^c^
PBS/APF	10/10 (100)	17 (13–35)	53/110 (48)	1,281 (428–12,410)

^a^P<0.05 by Mann-Whitney U test compared to corresponding PBS control group.

^b^P<0.05 by Fisher’s exact test compared to corresponding PBS control group.

^c^P>0.05 by Mann-Whitney U test compared to corresponding PBS control group.

^d^P>0.05 by Fisher’s exact test compared to corresponding PBS control group.

### Upper genital tract pathology: hydrosalpinx formation

To determine protection against long-term sequelae, at seven weeks following the vaginal challenge mice were euthanized and their genital tract macroscopically inspected. None of the mice had findings consistent with hydrosalpinx formation, suggesting limited upper genital tract pathology.

## Discussion

We have previously shown that phosphate substitution in the adjuvant used in a *C. trachomatis* Ser E rMOMP/SPA08 vaccine increased MOMP-specific IgG and decreased IFN-γ secretion in immunized mice.^32^ In the current study, we confirmed that as the P:Al molar ratio in the Ser E rMOMP/SPA08 vaccine formulations increased, the ELISA and neutralizing antibody titers also increased while the IFN-γ cellular response decreased. Hence, the formulation with the highest P:Al ratio, SPA08-4, compared with the other formulations, induced the highest neutralizing antibody GMT and the lowest IFN-γ cellular response. This formulation was associated with the lowest protection in the C. *trachomatis* vaginal challenge mouse model, thus, it would seem that high IFN-γ (Th1 response), rather than neutralizing antibodies, correlated better with protection in this model. The component within the vaccine that polarizes the rMOMP immunity toward an IFN-γ immune response profile and critically contributes to protection is the TLR4 agonist, E6020. This serves as a cautionary tale the fact that small changes within the formulation, unrelated to the concentration of antigen or TLR4 agonist, can turn a protective vaccine with a type I immune profile into a non-protective vaccine with a predominantly type II character, with far-reaching implications. The mechanisms by which phosphate substitution, protein adsorption and TLR-agonist adsorption influence the profile of the immune response against rMOMP are currently unknown. It is, however, plausible that these critical formulation parameters impact the residence time of the antigen and TLR-agonist at the site of injection, the uptake of the adjuvant – antigen complex by APCs and antigen presentation.^32,53^

The role of IFN-γ in clearance and protection against chlamydia infection is supported by evidence from both human and animal studies.^54-57^ Despite the vigorous antibody response to chlamydia infection or in vaccine models across different mammalian species, the role of antibodies is still poorly understood with some studies reporting protection and others a disease-enhancing effect.^58-60^ This is not surprising due to the huge heterogeneity of the antibody responses and our incomplete understanding of this critical component of the immune system. The results from *in vitro* experiments performed decades ago demonstrated that following a *C. trachomatis* infection, mice mounted humoral responses that resulted in the production of antibodies some of which were protective and others which had neutral or disease enhancing effects.^50,59,61^ The protective effect of monoclonal and polyclonal antibodies against MOMP has also been shown in passive transfer experiments.^58,62-64^ The only *C. trachomatis* vaccine undergoing evaluation in clinical trials is based on a polyvalent recombinant MOMP formulation that elicits high levels of neutralizing antibodies.^58,65,66^

The Ser E rMOMP/APF formulation induced a neutralizing antibody response characterized by high levels of total IgG, predominantly IgG1 subclass, and low levels of IFN-γ, suggestive of a type II immune response, primarily. However, it did not provide protection. This demonstrates that, in the present model, Ser E rMOMP antibodies with *in vitro* neutralizing capacity failed to protect against *C. trachomatis* Ser E challenge, therefore, the protection would appear to be dependent on an IFN-γ-mediated mechanism. The apparent discordance between the *in vivo* and *in vitro* results could be explained by the fact that the rMOMP antibodies exert their function via Fc receptors (FcRs) *in vivo* but that the FcRs are not necessary for *in vitro* neutralization. Also, the antibody isotype and the host cell type used for the *in vitro* neutralization assay can affect the results.^50,59,61^ Thus, this apparent negative effect is likely to be due to this particular vaccine preparation since protective results have been observed with rMOMP and native MOMP in other formulations.^21,26,65,67-69^ As seen with other recombinant vaccines, such as those for HBV and HPV, many of the *highly* protective B-cell epitopes are located in conformation-dependent domains. Thus, it may be that in this model the humoral immune protective responses are not fully achieved and hence play a secondary role to the cell-mediated immune responses.^70-73^ Future studies such as passive immunity transfer with different subclasses of rMOMP antibodies, could address this question to improve our understanding about which antibody-dependent mechanisms are protective and to de-risk further attempts to develop chlamydia vaccines.

The search for an effective *C. trachomatis* vaccine started more than a century ago, and although whole-organism vaccines were found to induce protective responses, their development stopped when enhanced disease was observed in some immunized individuals, a phenomenon known as vaccine-related immunopathology.^9,11,21,23,74,75^ Although this phenomenon is rare, it has been reported for other infectious diseases, such as RSV and measles.^76,77^ The rMOMP antigen, produced by recombinant technology in *E. coli*, is free from chlamydial contaminants that could be responsible for unwanted immune responses.

The efficacy of experimental vaccine formulations is tested in different animal models.^78,79^ To facilitate the interpretation of immunological responses the majority of vaccination experiments in animal models are performed in inbreed strains. This approach is extremely helpful for characterizing immune responses to vaccination and determining which correlate with protection. A limitation of using inbred animals is that they do not have the highly heterogeneous genetic diversity that humans have. The heterogeneous human genetic diversity means their response to the same immunological stimuli can vary. To mimic the heterogeneity better, we used outbred female CD-1 mice in this study. To our knowledge this is the first report of vaccine-induced protection in an outbred mouse strain against a vaginal challenge with *C. trachomatis*. This represents a significant step forward in chlamydia vaccine development. Previous reports have shown protection using homologous rMOMP in inbred mice challenged in the genital tract with *C. trachomatis* or *C. muridarum*.^65,67,80^

One limitation of the study is the lack of overt pathogenicity of the *C. trachomatis* serovar E in mice. The serovar E is a human pathogen with little affinity for murine cells and tissues which could explain its lack of pathogenicity. Moreover, *C. trachomatis* is generally not very virulent, even in humans. Infections are frequently asymptomatic, with overt pathogenicity in female upper genital tract typically detected only during severe acute infections or after years of chronic infection. Hence, reproducing *C. trachomatis* pathology in a time-contracted animal model is a challenge. The absence of hydrosalpinx formation, an indicator of upper genital tract pathology, confirms that none of the vaccinated or sham-vaccinated control mice developed long-term sequelae during the study. These results support the limited pathogenic nature of *C. trachomatis* in mice when delivered by the vaginal route.^81-83^

In conclusion, our results showed that the SPA08 vaccine formulation with no phosphate substitution provides better protection than formulations with phosphate substitution against a vaginal *C. trachomatis* challenge in mice, probably via a Th1 mechanism. Furthermore, it establishes the ability of a vaccine formulated with rMOMP to elicit protection in individuals with a broad diversity of genetic backgrounds.
